# Effect of Ambient Temperature on the Mechanical Properties of High Ductility Concrete

**DOI:** 10.3390/ma16062465

**Published:** 2023-03-20

**Authors:** Lijuan Chai, Bo Chen, Liping Guo, Biaokun Ren, Zhichun Chen, Tianyong Huang

**Affiliations:** 1 College of Civil Engineering, Taiyuan University of Technology, Taiyuan 030024, China; 2 State Key Laboratory of Solid Waste Reuse for Building Materials, Beijing 100041, China; 3 State Key Laboratory of Hydrology-Water Resources and Hydraulic Engineering, Nanjing Hydraulic Research Institute, Nanjing 210029, China; 4 School of Materials Science and Engineering, Southeast University, Nanjing 211189, China; 5 Technical Supervision and Research Center of the Building Materials Industry, Beijing 100024, China

**Keywords:** high ductility concrete (HDC), temperature, compressive performance, tensile performance, flexural performance, pore structure

## Abstract

This study analyzes the mechanical properties of high ductility concrete (HDC) under different ambient temperatures to provide a parameter basis for the design of HDC bridge deck link slabs. Five temperatures (−30, 0, 20, 40, and 60 °C) were designed to investigate the compressive, tensile, and flexural properties of HDC after temperature treatment and analyze the pore structure. The results show that, compared with the HDC performance at room temperature (20 °C), the compressive strength, ultimate tensile strength, and flexural strength decreased after treatment at low temperatures (−30 and 0 °C), while the strength increased after treatment at high temperatures (40 and 60 °C). After experiencing low- and high-temperature treatments, the ultimate tensile strain and ultimate deflection of the HDC increased. The tensile and flexural failures of the HDC exhibited multiple cracking, and the stress–strain/deflection curve showed a strain/deflection hardening stage. The tensile constitutive relationship can be simplified as a bilinear two-stage relationship. As the temperature increased, the porosity of harmless and less harmful pores in HDC gradually increased, while the porosity of harmful and more harmful pores gradually decreased, resulting in an increase in HDC strength. Based on the influence of temperature on HDC properties, design parameters for the HDC bridge deck link slab structure are proposed.

## 1. Introduction

**Figure 1:**
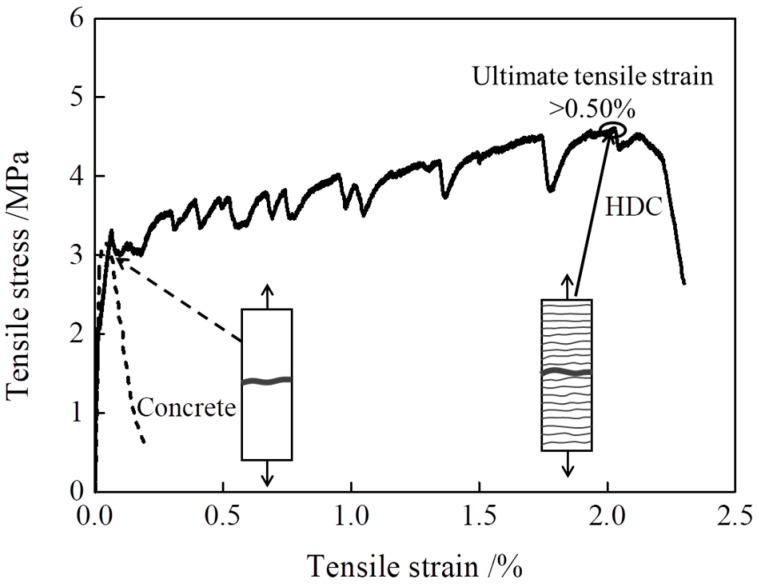
Schematic diagram of tensile stress–strain relationship curve and multiple cracking for HDC.

High ductility concrete (HDC) is a type of concrete that uses fibers as reinforcement material and is designed at multiple scales, from the microscale to the macroscale. Under uniaxial tension, HDC exhibits multiple cracking characteristics and an ultimate tensile strain not less than 0.50%, with an average crack width not exceeding 200 μm, as shown in Figure 1 [1]. HDC has an excellent tensile deformation capacity under uniaxial tension and can be used in engineering applications with high flexural and tensile deformation requirements [2,3,4].

**Figure 2:**
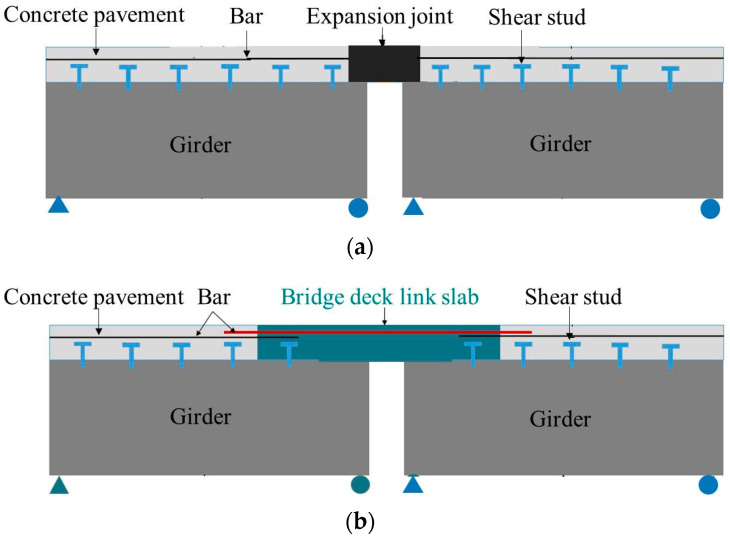
Schematic diagram of expansion joint and bridge deck link slab. (**a**) Expansion joint. (**b**) Bridge deck link slab.

HDC material can be cast into bridge deck link slabs to replace expansion joints in bridge engineering, making the entire bridge deck continuous and seamless, as shown in Figure 2. The pilot project monitoring results of HDC bridge deck link slabs show that HDC maintains excellent performance during the service life of the bridge [5,6], indicating that HDC bridge deck link slabs have promising application prospects.

China has a significant difference in regional temperatures, with the ground temperature in the northeastern region reaching around −30 °C in winter, around 0 °C in the central region, and up to 60 °C in the southern region during summer. Bridge deck link slabs are exposed to natural environments and are influenced by regional temperatures. The mechanical property of fiber-reinforced concrete is affected by the temperature curing condition, as the properties of fibers, the matrix, and the interface between the matrix and fibers will vary [7,8,9]. HDC is a kind of fiber-reinforced concrete, and the mechanical property of HDC is also influenced by the ambient temperature, which will affect the key design parameters of the mechanical properties of HDC bridge deck link slabs. Some researchers have analyzed the mechanical properties of HDC exposed to different temperatures [10,11,12]. Yu et al. [10] found that the ultimate tensile strength and ultimate tensile strain of PVA fiber-HDC specimens increased after standard curing for 28 days and then subjected to 50 and 100 °C for one hour. Dong et al. [11] standard-cured PE fiber-HDC specimens for 28 days and then performed compressive and tensile tests at −20, 0, 30, 60, and 90 °C. Within the range of −20 °C to 90 °C, as the temperature increased, the compressive strength, peak compressive strain, and ultimate tensile strength of HDC gradually decreased, while the ultimate tensile strain gradually increased. Li et al. [12] analyzed the compressive and tensile properties of HDC exposed to curing temperatures of −20, 15, and 20 °C. The research results revealed that with the increasing curing temperature, the compressive strength and splitting tensile strength of HDC increased gradually. PVA fibers are hydrophilic, and PE fibers are hydrophobic. The ultimate elongation of PE fibers is larger than that of PVA fibers, and the sliding mechanism of PE fibers in the matrix is different from that of PVA fibers, resulting in differences in the mechanical properties of HDC prepared with the two types of fibers [13,14]. Currently, the mechanical properties of HDC prepared with PVA fibers in a natural environment ranging from −30 °C to 60 °C are rarely reported.

In this study, the HDC material was prepared first. Then, taking HDC bridge deck link slabs exposed to the natural environment as the engineering background, the compressive, tensile, and flexural properties of the HDC material after curing at −30, 0, 20, 40, and 60 °C were analyzed. Mercury intrusion porosimetry was used to analyze the pore structure to reveal the macroscopic performance mechanism. This study can provide material performance parameters for the structural design of HDC bridge deck link slabs in different regions.

## 2. Materials and Methods

### 2.1. Raw Materials and Mix Proportions

The cementitious materials used in this study were P.II42.5R cement (C) and Class II fly ash (FA). The aggregate was river sand (S) with a maximum particle size of 1.18 mm, a fineness modulus of 1.68, and a particle density of 1605 kg/m^3^. The water reducer used was a powdered polycarboxylate superplasticizer (PS), and tap water (W) was used as the mixing water. The PVA fibers used in the experiment were domestically produced, with a length of 12 mm, an average diameter of 39μm, an elastic modulus of 30 GPa, an ultimate elongation of 5~8%, and an ultimate tensile strength of 1250 MPa [15].

To ensure that the strength grade of HDC bridge deck link slabs is consistent with the adjacent concrete pavement layer, the design strength level of concrete bridge deck link slabs should be no less than C40 based on the bridge code [16]. Therefore, the mix proportion of HDC was designed to be C40, as shown in Table 1 [15].

### 2.2. Specimen Preparation Process

**Figure 3:**
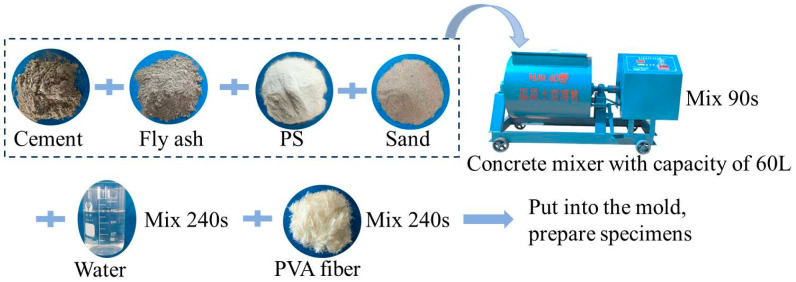
Preparation process of HDC.

The preparation of HDC materials was carried out using a 60 L concrete mixer. The main steps of the preparation process were as follows: (1) mix the solid materials cement, fly ash, water reducer, and river sand in the mixer for 90 s; (2) add water and continue mixing for 240 s; (3) add fibers and mix for an additional 120 s; and (4) pour the mixture into the mold, compact it, cover the surface of the specimen with plastic film and let it stand at (20 ± 2) °C for 1 day and then cure the specimen at (20 ± 2) °C for 27 days. The steps for preparing the HDC specimens are shown in Figure 3.

### 2.3. Test Methods

#### 2.3.1. Optimization of HDC Mix Proportion

The objective of the HDC mix proportion optimization is to achieve a compressive strength grade of at least C40 and an ultimate tensile strain of at least 0.50%. The dimensions of the cubic compressive strength test specimens and the uniaxial tension test specimens used in the optimization of the HDC mix proportion are shown in Table 2 [1].

**Figure 4:**
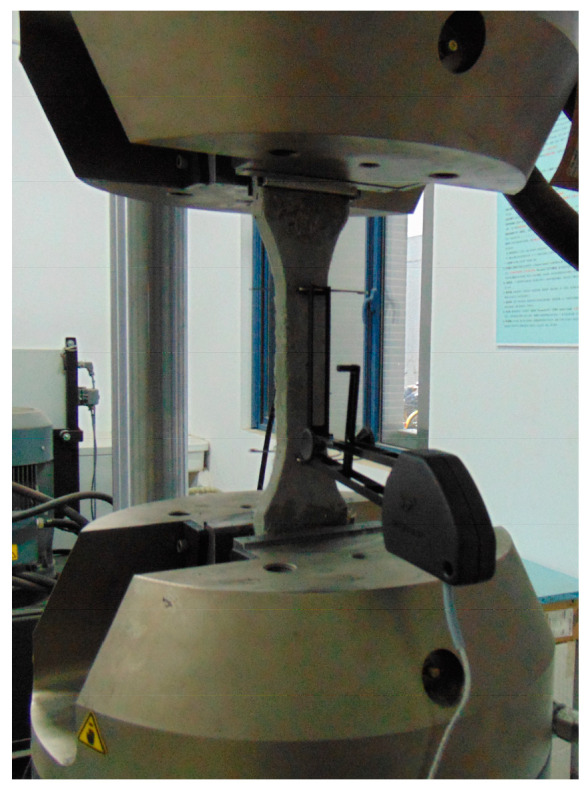
Test device for tension.

The cubic compressive strength of HDC test specimens was measured using an electro-hydraulic servo pressure testing machine. The loading mode for the cubic compressive strength test was load-controlled at a rate of 0.5 MPa/s. The uniaxial tension performance test of HDC dog-bone specimens was conducted using an MTS (materials testing system)-810 tensile testing machine. The loading mode for the uniaxial tension test was displacement-controlled at a rate of 0.2 mm/min. An LDVT (linear variable displacement transducer) was used to measure the elongation of the specimen during the tension test in the middle 100 mm region of the dog-bone specimen, as shown in Figure 4.

#### 2.3.2. Fiber Dispersion Method

**Figure 5:**
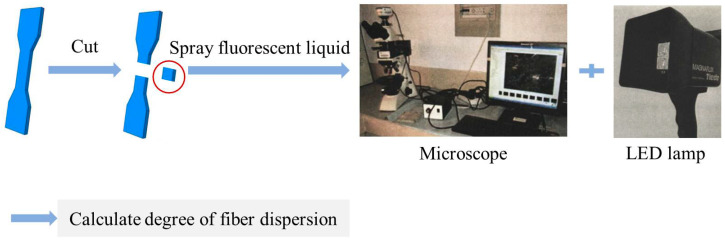
Test method flow of fiber dispersion degree.

Based on the optimal proportioning of HDC, the fiber dispersion test was conducted. A small prism specimen with dimensions of 13 mm × 30 mm × 10 mm was cut out from the center of the tensile specimen using a cutting machine. The cutting surface (13 mm × 30 mm) was sprayed with a fluorescent liquid water spray and allowed to stand for 10~15 min. The excess fluorescent liquid on the cutting surface was washed off with water, and the specimen was left to dry naturally. The specimen was then placed under a polarizing microscope for observation and illuminated with an LED ultraviolet light source. A total of 60 photos were taken for each specimen using the CCD camera built into the microscope. The PVA organic fibers can absorb the fluorescent liquid water spray, and under LED ultraviolet light, the fibers appear light blue. The fiber dispersion of PVA fibers can be calculated from the images [17,18,19]. The test method process is shown in Figure 5.

#### 2.3.3. Mechanical Property Testing of HDC after Different Temperature Treatments

Specimens for mechanical property testing of HDC were prepared based on the optimal proportioning of HDC. The sizes of the specimens used for compressive, tensile, and flexural property testing of HDC are shown in Table 3 [1].

After the specimens were cured for 28 days, they were kept at a constant temperature of −30, 0, 20, 40, and 60 °C for 8 h in order to make the whole HDC specimen suffer the temperature sufficiently. Then, specimens were allowed to rest at room temperature (20 ± 2) °C for 1 h before mechanical testing.

**Figure 6:**
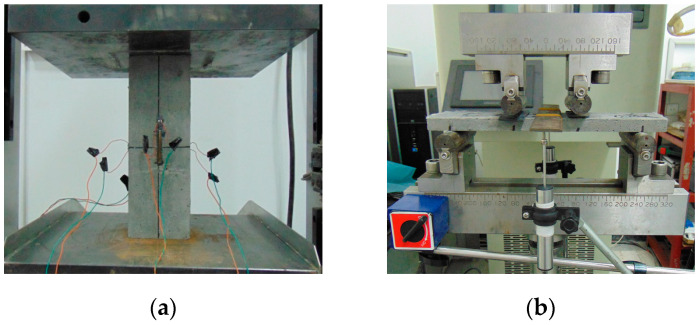
Test setup. (**a**) Axial compressive test. (**b**) Flexural test.

The axial compressive performance of HDC specimens was tested using an electro-hydraulic servo pressure testing machine, with a loading mode of displacement control and a speed of 0.3 mm/min [20]. Strain gauges of the BX120-50AA type were symmetrically attached to the longitudinal and transverse sections in the middle third of the HDC specimens, which approximately resembled the uniaxial compression section. The elastic modulus and Poisson’s ratio of HDC were calculated based on the strain values [20]. The test device for the axial compressive performance of HDC specimens is shown in Figure 6a. The uniaxial tensile performance of HDC specimens was tested using the same device as shown in Figure 4. The flexural performance of HDC specimens was tested using the INSTRON 8802 electro-hydraulic fatigue testing machine, with a loading speed of 0.2 mm/min and the flexural deflection measured by an LDVT [21]. The test device for the flexural performance of HDC specimens is shown in Figure 6b.

#### 2.3.4. Microscopic Performance Testing

The Autopore IV9510 automatic mercury porosimeter was used to analyze the pore structure of HDC at different temperatures.

## 3. Results and Discussion

### 3.1. Optimal Proportioning of HDC

**Figure 7:**
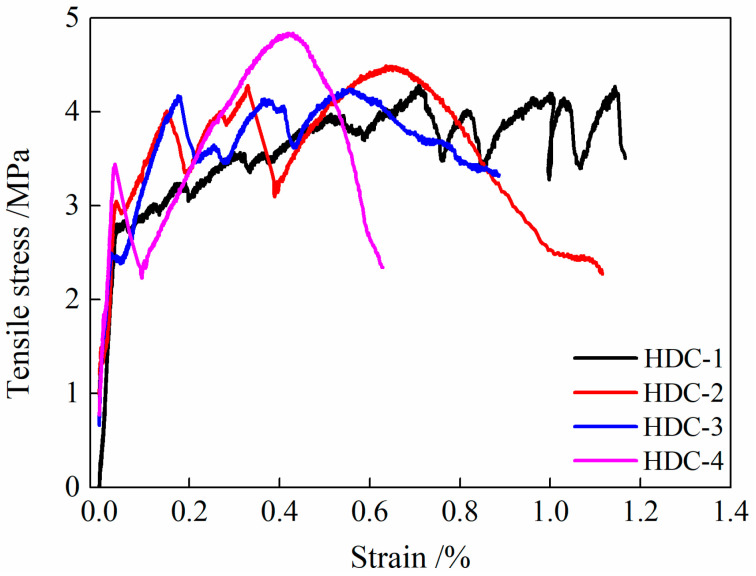
Tensile stress–strain relationship curves of HDC with different mixtures.

The compressive strength, ultimate tensile strength, and ultimate tensile strain of the four HDC mix proportions designed in this study are shown in Table 4, and the stress–strain curves under tension are shown in Figure 7. Notably, for each HDC specimen group with six curves, a representative curve is selected for easy comparison. Of the six curves, the one that has the ultimate tensile strain closest to the corresponding calculation value (in Table 4) will be selected as the representative curve.

From Table 4 and Figure 7, it can be seen that HDC-1 has the best compressive and tensile performance, as well as the greatest ultimate tensile strain. In the HDC-1 mix proportion, a large amount of fly ash is used, which, according to traditional HDC design theory [5], can reduce the fracture toughness of the matrix and the interfacial bond strength between the fibers and the matrix. During the tensile process of HDC, more fibers gradually pull out, fully exerting the fiber-bridging effect, and the HDC can achieve stable strain hardening, showing the superior ultimate tensile strain. Based on these four mix proportions, HDC-1 was selected for subsequent research on the mechanical properties after temperature treatment.

### 3.2. Fiber Dispersion Property

**Figure 8:**
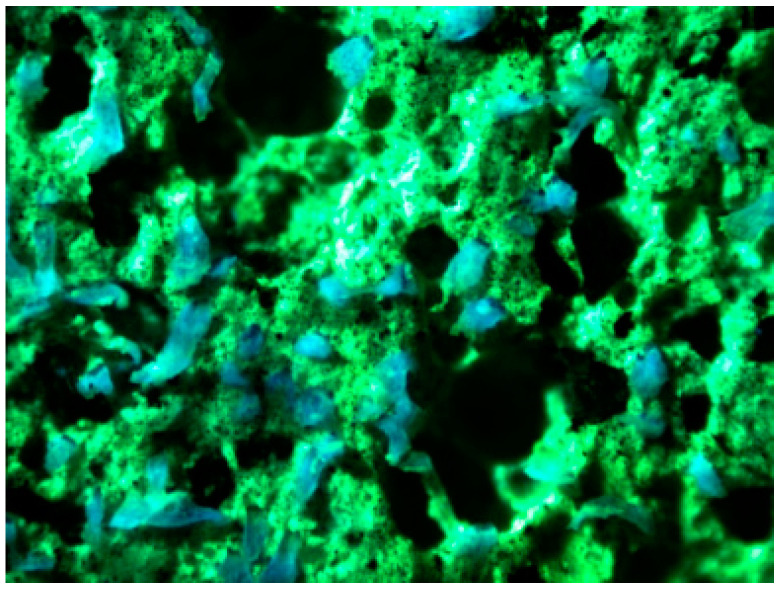
Fiber dispersion image.

Based on the optimal mix proportion, the fiber dispersion of HDC was tested, as shown in Figure 8. The light blue represents PVA fibers, and the green represents the remaining fluorescent liquid in the matrix. The fiber dispersion can be quantitatively evaluated by the number of PVA fibers in each image, and the calculation of the fiber dispersion coefficient (α) is shown in the following equation [17,18]:

(1) $$\alpha =\exp\left(-\sqrt{\frac{\Sigma {\left(x_{i}/x_{a}-1\right)}^{2}}{n}}\right)$$

where α represents the fiber dispersion coefficient, $x_{i}$ represents the fiber quantity in the *i*-th image, $x_{a}$ represents the average fiber quantity in all images, and n represents the number of images.

Using quantitative calculation, the fiber dispersion coefficient of HDC is 0.91, which is close to 1, indicating that the fiber dispersion of the specimens prepared by the mix proportion used in the experiment is good. Fiber dispersion is closely related to material mix proportion. The addition of fly ash can increase the ζ potential in HDC slurry, and the stronger repulsive force between particles makes it easier to disperse the particles, resulting in a more uniform matrix structure and improving fiber dispersion [22,23]. If the particle size of the aggregate is too large, it will cause fiber agglomeration. In the experiment, river sand with a maximum particle size of 1.18 mm was selected to promote uniform fiber dispersion and ultimately result in good fiber dispersion in HDC.

### 3.3. Compressive Performance

#### 3.3.1. Failure Mode of Prism

**Figure 9:**
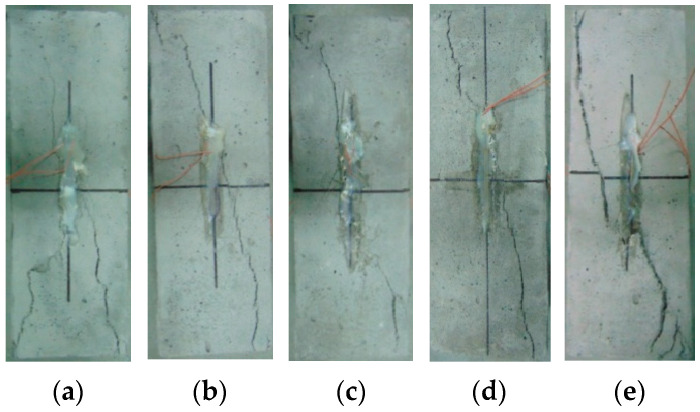
Compressive failure mode of HDC exposed to different temperature treatments. (**a**) −30 °C. (**b**) 0 °C. (**c**) 20 °C. (**d**) 40 °C. (**e**) 60 °C.

The failure modes of HDC prisms after different temperature treatments are shown in Figure 9. The final failure mode of all HDC specimens was consistent, showing a main diagonal crack passing through the entire cross-section of the specimen. The 150 mm height range in the middle of the prism specimens is under approximately uniaxial compression, and the top and bottom ends of the specimens are constrained by the loading pads to form a confining effect. When the HDC specimens are subjected to a load, cracks first appear at the weak middle part of the specimens, and with the increase of applied load, the cracks develop diagonally upward and downward from the middle of the specimens, forming diagonal cracks. The diagonal crack bands gradually widen, and finally, the main crack passing through the entire cross-section of the HDC specimens is formed when they fail.

#### 3.3.2. Characteristic Parameters

**Figure 10:**
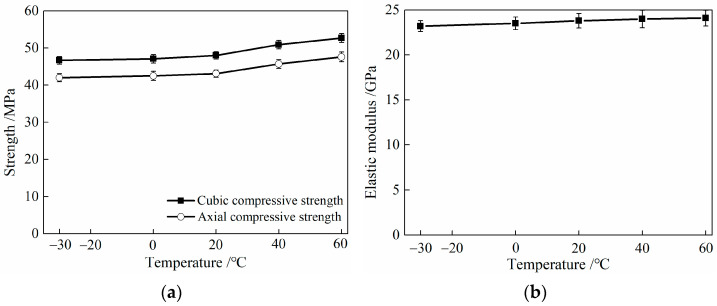
Relationship between feature parameters of compressive property and temperature for HDC. (**a**) Strength. (**b**) Elastic modulus.

The experimental results of the compressive strength test of HDC after different temperature treatments are shown in Table 5 and Figure 10. The cubic compressive strength, axial compressive strength, and elastic modulus of HDC decrease with decreasing temperature after low-temperature treatment (−30 and 0 °C), and the reduction in performance indicators is greater with lower temperatures. After high-temperature treatment (40 and 60 °C), the cubic compressive strength, axial compressive strength, and elastic modulus of HDC show an increasing trend, and the rate of increase in performance indicators is greater with higher temperatures.

Temperature treatment can affect the internal pore structure distribution of HDC. The low-temperature treatment of HDC causes water to freeze, which slows down the hydration rate of the cementitious material on the one hand and increases the porosity of large internal pores on the other hand, resulting in a loose structure of HDC material and a decrease in its cubic compressive strength, axial compressive strength, and elastic modulus [12]. Although free water evaporates from HDC during high-temperature treatment, high temperatures accelerate the hydration process of un-hydrated cement or fly ash [24,25], resulting in an increase in the strength and elastic modulus of HDC specimens. 

As shown in Table 5, the Poisson’s ratio of HDC specimens is 0.23 for both low- and high-temperature treatments. The conversion coefficient between the axial compressive strength and the cubic compressive strength of HDC is 0.90, whereas the ratio between the axial compressive strength and the cubic compressive strength of ordinary concrete is 0.76 [26]. HDC material does not contain coarse aggregate, and the maximum particle size of fine aggregate is 1.18 mm. The strength of HDC is less affected by the particle size of aggregate, resulting in a larger strength conversion coefficient for different size tests than that of ordinary concrete materials. From Table 5, it can be seen that the strength conversion coefficient of HDC after different temperature treatments is 0.90, indicating that the strength size effect coefficient is not significantly affected by temperature.

#### 3.3.3. Structural Design Parameters

Although the cubic compressive strength, axial compressive strength, and elastic modulus of HDC decrease after low-temperature −30 °C treatment, the degree of reduction is less than 3%. HDC bridge deck link slabs mainly bear tensile deformation caused by temperature differences and are not significantly affected by compressive strength. In the structural design of bridge deck link slabs, the effect of temperature on HDC compressive strength can be ignored, and the compressive performance indicators of HDC at normal temperatures can be directly used. The stress–strain constitutive relationship of HDC under compressive stress at normal temperatures can be referenced from the literature [27].

### 3.4. Tensile Performance

#### 3.4.1. Failure Mode

**Figure 11:**
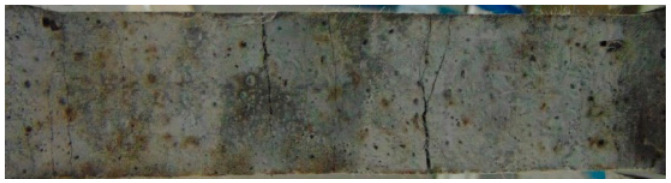
Multiple cracking pattern of tensile specimen for HDC.

After being treated at different temperatures, the HDC tensile specimens showed multiple cracking patterns, as shown in Figure 11. As the cracks in all specimens were small and difficult to observe with the naked eye, multiple cracking failure modes are shown in Figure 11 using the cracking pattern of HDC at a normal temperature as an example. In the initial loading stage, the HDC matrix bears the load. When the external load exceeds the tensile strength of the matrix itself, the matrix cracks, and the tensile stress is borne by the bridging fibers across the crack, which then transfers the tensile stress to the surrounding uncracked matrix. The uncracked matrix can continue to bear the load. Under the repeated action of “matrix bearing load-matrix cracking-fiber bridging-matrix bearing load-matrix cracking…”, HDC specimens exhibit multiple cracking patterns, which is an intrinsic characteristic of HDC material and is not related to the temperature treatment method. As the applied load increases, the number of cracks in the HDC specimen increases, and eventually, the cracks deteriorate and widen, forming the main crack.

#### 3.4.2. Tensile Stress–Strain Relationship Curve

**Figure 12:**
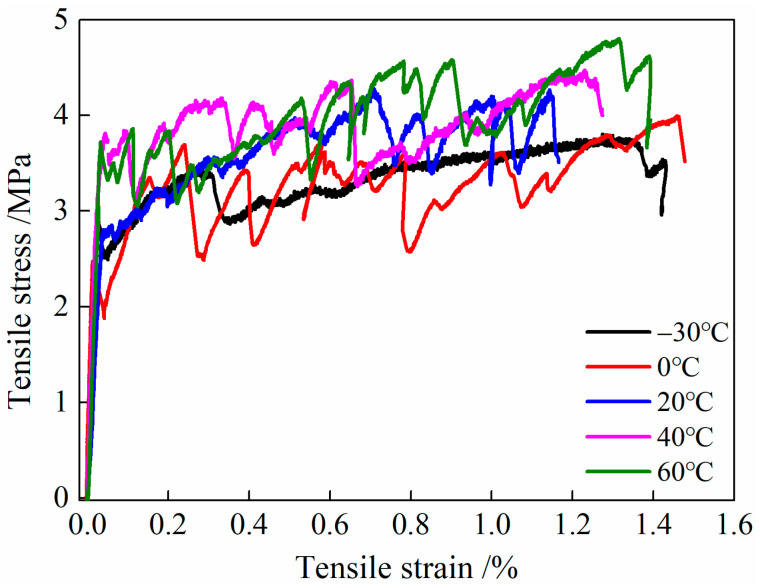
Tensile stress–strain relationship curve of HDC.

The uniaxial tensile performance curve of HDC after different temperature treatments is shown in Figure 12. Similar to the selection principle of the representative tensile stress–strain relationship curve of HDC in Figure 7, the representative curve of HDC with different temperature treatments in Figure 12 is determined based on the curve that has the ultimate tensile strain closest to the corresponding calculation value (in Table 6).

From Figure 12, we can see the tensile stress–strain relationship curves of HDC after different temperature treatments all show strain-hardening phenomena. In the strain-hardening stage of HDC from 0 to 60 °C, there is a significant “down-up-down” oscillation trend in stress, but after low-temperature (−30 °C) treatment, the strain-hardening stage of HDC exhibits a relatively smooth trend without significant oscillation characteristics.

After undergoing different temperature treatments, the uniaxial tensile performance curves of HDC display strain hardening, which is an inherent characteristic of the material and is independent of the environmental conditions. During the initial loading stage, the HDC matrix bears the tensile force. As the load increases, the HDC matrix cracks, resulting in a decrease in the stress of the tensile performance curve. The fibers then carry the bridging stress and transfer it to the surrounding uncracked matrix, which can continue to bear the load, causing the stress in the curve to increase. Once the tensile load exceeds the matrix tensile strength, the matrix cracks, and the fibers carry the bridging stress, resulting in the curve displaying a strain-hardening phenomenon [28]. The strain-hardening characteristics of HDC are related to the properties of the matrix, fibers, and fiber/matrix interface. After undergoing low-temperature treatment at 0 °C, the HDC matrix becomes loose, the tensile strength of the fibers decreases, and the chemical adhesion stress between the fibers and the matrix decreases. At the same time, the frictional stress at the interface increases, which helps the fibers to slowly pull out of the matrix and fully exert their bridging effect [29]. This leads to a distinct stress “drop-rise-drop” jitter trend in the curve. However, when subjected to low-temperature treatment at −30 °C, the strength loss of the matrix and fibers is relatively high, and their carrying capacity is relatively low. Internal damage exists within the specimen, and under external load, the internal damage gradually deteriorates, causing the stress to gradually decrease. As a result, the curve shows a relatively “smooth” state without any significant jitter characteristics. After undergoing high-temperature treatments of 40 and 60 °C, the strength of the matrix increases, the strength of the fibers decreases, and the chemical adhesion stress at the fiber/matrix interface decreases. At the same time, the frictional stress at the interface increases, which helps to fully exert the bridging effect of the fibers [29]. This leads to a distinct jitter trend in the curve.

#### 3.4.3. Characteristic Parameters

**Figure 13:**
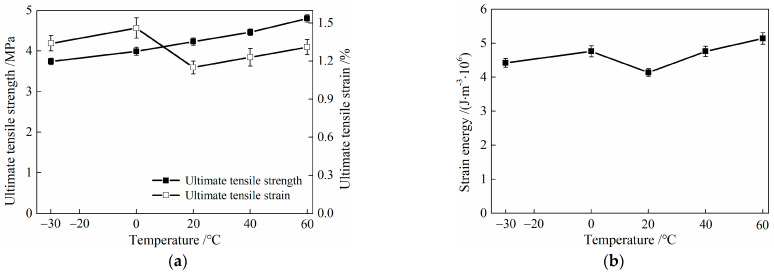
Relationship between feature parameters of tensile property and temperature for HDC. (**a**) Ultimate tensile strength and ultimate tensile strain. (**b**) Strain energy.

After low- and high-temperature treatments, the characteristic parameters of the tensile performance of HDC are shown in Table 6 and Figure 13. Strain energy in Table 6 refers to the area under the stress–strain curve of HDC during tension, with the abscissa ranging from the initial tensile strain point to the ultimate tensile strain point. Strain energy can reflect the energy consumption of HDC material after experiencing different temperature treatments. 

Table 6 and Figure 13 show that compared with HDC material at room temperature (20 °C), the ultimate tensile strength of HDC decreases after low-temperature (−30 and 0 °C) treatment, while it shows an increasing trend after high-temperature (40 and 60 °C) treatment. The greater the amplitude of the temperature decrease or increase, the greater the change in ultimate tensile strength. After low- and high-temperature treatments, the ultimate tensile strain and strain energy of HDC show an increasing trend. The greatest increase in ultimate tensile strain occurs after low-temperature treatment at 0 °C, and the greatest increase in strain energy occurs after high-temperature treatment at 60 °C.

The ultimate tensile strength and ultimate tensile strain of HDC are related to the properties of the HDC matrix, PVA fibers, and fiber/matrix interface. The impact of the fiber-bridging ability on the ultimate tensile strain of HDC is greater than that on the ultimate tensile strength. After low-temperature (−30 and 0 °C) and high-temperature (40 and 60 °C) treatments, the chemical bonding stress between the fibers and matrix interface decreases, while the interface frictional stress increases. Among them, the chemical bonding stress between the fibers and matrix decreases the most after the 0 °C treatment [29]. The decrease in the fiber–matrix interface bonding stress and the increase in the interface frictional stress can both improve the fiber-bridging stress [29,30]. Although the fiber-bridging stress in HDC increases after low-temperature treatment [31], the strength of the matrix and fibers will weaken, resulting in a decrease in the ultimate tensile strength of HDC but an increase in its ultimate tensile strain. After the 0 °C low-temperature treatment, the improvement effect of fiber-bridging stress is the best, resulting in the largest increase in the ultimate tensile strain of HDC. After high-temperature treatment, the fiber-bridging ability and matrix strength of HDC both increase, leading to an increase in both the ultimate tensile strength and ultimate tensile strain of HDC. The strain energy of HDC is related to the tensile stress and strain. After low-temperature and high-temperature treatments, the ultimate tensile strain of HDC increases, and the increase is greater than that of the strength, resulting in an overall increase in strain energy.

#### 3.4.4. Tensile Stress–Strain Relationship Model

**Figure 14:**
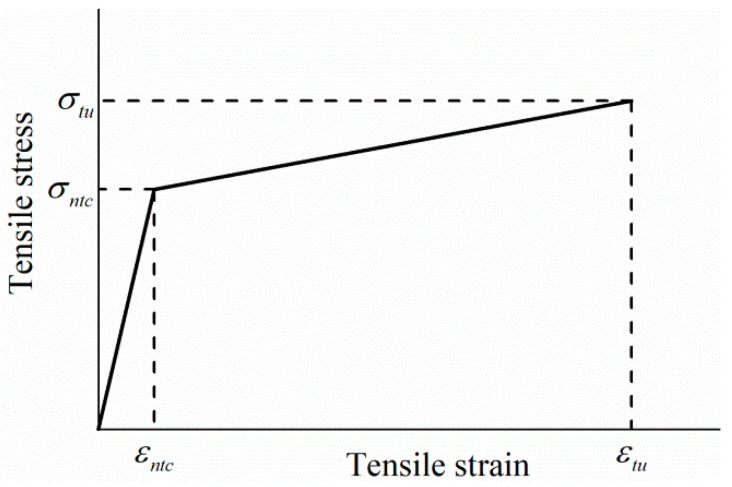
Simplified two-stage model of tensile stress–strain relationship for HDC.

Establishing the tensile stress–strain relationship model of HDC can provide theoretical parameters for the design of HDC bridge deck link slab structures. The initial cracking point in the tensile stress–strain curve of HDC is mainly related to the matrix properties. As the matrix has low tensile strength and deformation, it is difficult to accurately measure the initial cracking tensile strength and initial cracking strain. Therefore, the nominal initial cracking tensile strength $\sigma_{ntc}$ [32] is introduced, which is defined as the intersection point between the strain-hardening stage data fitting curve and the straight line in the linear elastic stage, as shown in Figure 14.

Based on the characteristics of the HDC tensile stress–strain curve, it is simplified into a two-stage model, with the first stage being the elastic stage and the second stage being the strain-hardening stage, which is a simplified linear segment. $\varepsilon_{ntc}$ represents the nominal initial cracking strain corresponding to the nominal initial cracking tensile strength $\sigma_{ntc}$.

The calculation of the two-stage model for the HDC tensile performance curve is shown in the following equation:

(2) $$\sigma (\varepsilon )=E_{tc}\cdot \varepsilon \;\varepsilon \leq \varepsilon_{ntc}$$

(3) $$\sigma (\varepsilon )=\sigma_{ntc}+E_{tu}\left(\varepsilon -\varepsilon_{ntc}\right)\;\varepsilon_{ntc}<\varepsilon \leq \varepsilon_{tu}$$

where $\sigma \left(\varepsilon \right)$ is the tensile stress (MPa), ε is the tensile strain, $E_{tc}$ is the initial elastic modulus (MPa) determined by fitting the experimental curve data points, and the fitting range is from 0 to the onset of cracking. $E_{tu}$ is the strain-hardening elastic modulus (MPa), with the “lower yield point” of the yield stage of the tensile stress–strain curve of low carbon steel as the design strength, determined by fitting the “valleys” of the fluctuation segment in the strain-hardening stage of the HDC test curve with the fitting range from the onset of cracking to the ultimate point.

According to the HDC tensile stress–strain test data and Formulas (2) and (3), the key parameters of the two-stage model are calculated, as shown in Table 7, where $R^{2}$ represents the correlation fitting degree of $E_{tc}$ and $E_{tu}$.

#### 3.4.5. Structural Design Parameters

After low-temperature treatment at −30 °C, the maximum reduction in the ultimate tensile strength of HDC is observed, while the ultimate tensile strain and strain energy both increase. After high-temperature treatment, both the ultimate tensile strength and ultimate tensile strain of HDC increase. In the design of the HDC bridge deck link slab structure, it is necessary to consider the influence of temperature on the tensile properties of HDC materials and to use a simplified two-stage model based on the HDC tensile stress–strain constitutive relationship for structural design, according to the regional temperature characteristics.

### 3.5. Flexural Performance

#### 3.5.1. Failure Mode

**Figure 15:**
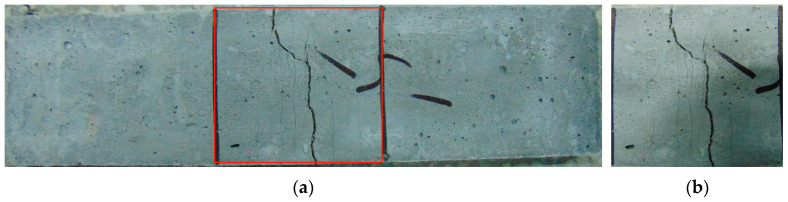
Multiple cracking pattern of flexural specimen for HDC. (**a**) Whole specimen. (**b**) Pure flexural zone.

After undergoing different temperature treatments, the flexural specimen of HDC shows a multiple cracking mode in the pure bending section, as shown in Figure 15. Some individual micro-cracks are difficult to observe with the naked eye, and the flexural failure mode of HDC is similar to the tensile failure mode. In the initial loading stage, the HDC matrix bears the load. After the matrix cracks, the cross-seam fibers bear the flexural and tensile stresses and transfer the stresses to the surrounding uncracked matrix, which continues to bear the load. Under the combined action of the matrix and fibers, HDC exhibits a multiple cracking mode, which is independent of temperature and is the inherent feature of HDC material. As the load increases, the number of cracks increases, and the cracks deteriorate and form the main crack. Due to the bridging effect of randomly distributed fibers, the cracks are not straight, and the cracks deflect [33].

#### 3.5.2. Flexural Stress–Deflection Relationship Curve

**Figure 16:**
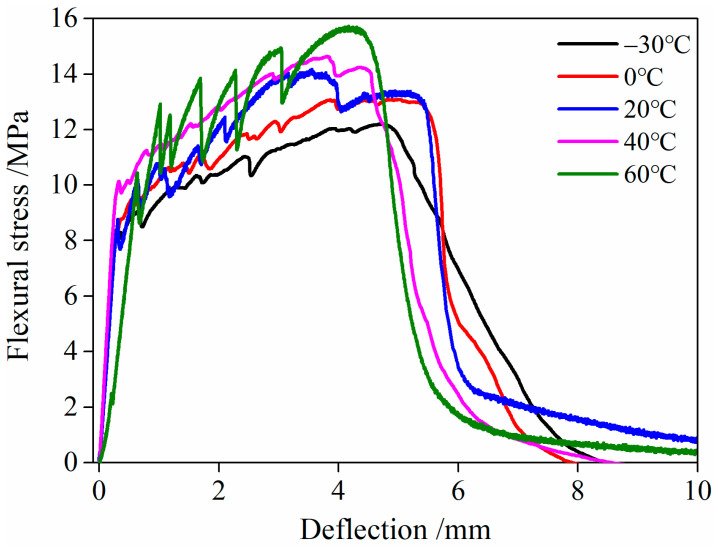
Flexural stress and deflection relationship curve of HDC.

The flexural stress–deflection relationship curve of HDC after undergoing different temperature treatments is shown in Figure 16. Among the three tested cures, the representative curve in Figure 16 is screened based on the curve that has the ultimate deflection closest to the corresponding calculation value (in Table 8).

As presented in Figure 16, after undergoing different temperature treatments, the flexural stress–deflection relationship curve of HDC exhibits strain hardening, which is similar to the uniaxial tensile stress–strain relationship curve. In the initial loading stage, the HDC matrix bears the load. After the applied load exceeds the flexural strength of the matrix, the matrix cracks, and the transverse fibers across the crack bear the stress and play a bridging role in transferring the load to the uncracked matrix, allowing HDC to continue to bear the load. Under the action of “matrix cracking-fiber bridging-matrix cracking…”, the flexural stress–deflection relationship curve of HDC exhibits the characteristic of “decreasing-increasing-decreasing”, which is the essential mechanism of the high ductility of HDC material and is independent of the curing environment temperature and loading mode. Therefore, the flexural stress–deflection relationship curve of HDC and the uniaxial tensile stress–strain relationship curve both exhibit the characteristic of strain/strain hardening.

#### 3.5.3. Characteristic Parameters

**Figure 17:**
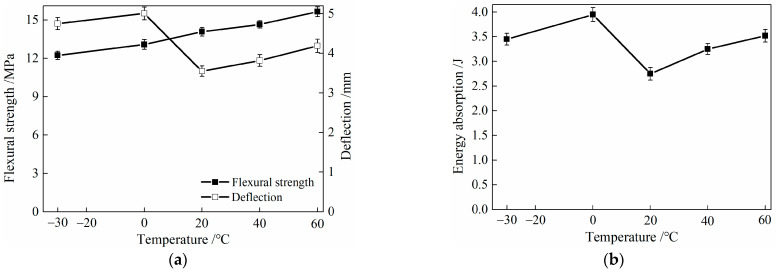
Relationship between feature parameters of flexural property and temperature for HDC. (**a**) Flexural strength and deflection. (**b**) Energy absorption.

After undergoing low- and high-temperature treatments, the characteristic parameters of the flexural performance of HDC are shown in Table 8 and Figure 17. The energy absorption value in the table refers to the area under the flexural load–deflection relationship curve of HDC, and the abscissa of the area calculation ranges from the initial mid-span deflection to the ultimate mid-span deflection.

From Table 8 and Figure 17, it can be observed that the ultimate load and flexural strength of HDC decrease after low-temperature (−30 and 0 °C) treatment compared to its performance at room temperature (20 °C). However, the ultimate deflection and energy absorption values of HDC increase after low-temperature treatment. On the other hand, after high-temperature (40 and 60 °C) treatment, the ultimate load, flexural strength, ultimate deflection, and energy absorption values of HDC increase. The ultimate deflection and energy absorption values of HDC are the highest after being treated at a low temperature of 0 °C. 

Similar to the uniaxial tension performance, the effect of fiber-bridging ability on the ultimate deflection deformation capacity of HDC is greater than its effect on strength. The bonding stress at the fiber/matrix interface in HDC decreases, while the interface friction stress increases after low- and high-temperature treatments, which improves the fiber-bridging ability and increases the ultimate deflection of HDC at mid-span. The most significant improvement in fiber-bridging ability is observed when HDC is treated at a low temperature of 0 °C, resulting in the largest increase in the ultimate deflection of HDC. After low-temperature treatment, the strength of the HDC matrix and fibers deteriorates, leading to a decrease in the ultimate load and flexural strength of HDC. Conversely, high-temperature treatment can increase the strength of the HDC matrix, resulting in an increase in the ultimate load and flexural strength of HDC. The energy absorption value is related to the load and deflection values, which is the area under the flexural load–deflection curve. The most significant increase in the ultimate deflection is observed after low- and high-temperature treatments, leading to a consistent change in the energy absorption value and ultimate deflection. 

After low-temperature treatment, the flexural strength of HDC decreases, and the ultimate deflection increases, while after high-temperature treatment, both the flexural strength and ultimate deflection of HDC increase. Therefore, the effect of temperature on the flexural performance parameters of HDC materials should be considered in the analysis of the structural performance of HDC bridge deck link slabs.

### 3.6. Pore Structure

**Figure 18:**
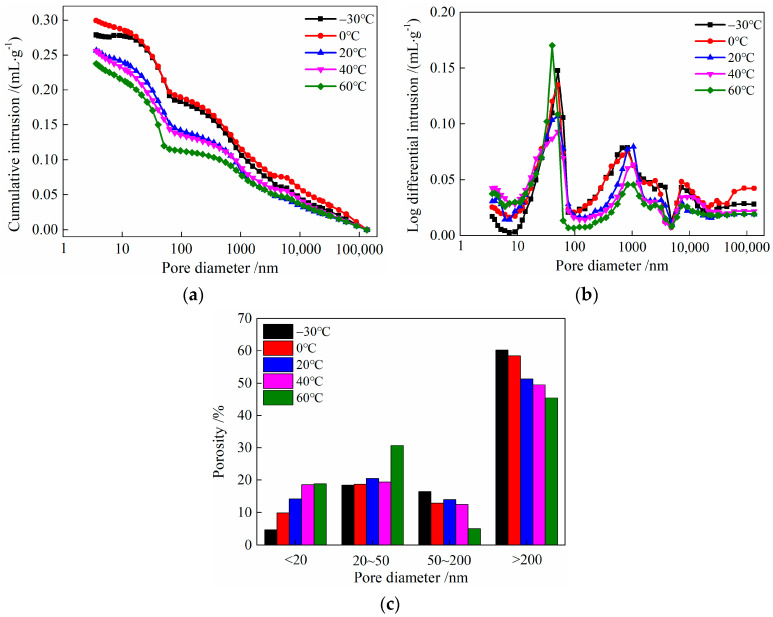
Pore size distribution of HDC. (**a**) Cumulative intrusion curve. (**b**) Log differential intrusion curves. (**c**) Porosity.

The pore structure of HDC after undergoing different temperature treatments is shown in Figure 18. As shown in Figure 18a, the critical pore size of HDC after normal curing is 62.4 nm. After −30, 0, and 40 °C treatments, the critical pore size of HDC does not change significantly, while after a 60 °C treatment, the critical pore size of HDC is 50.3 nm. As shown in Figure 18b, after −30, 0, 20, and 40 °C temperature treatments, the most probable pore size of HDC is 50.3 nm, while after a 60 °C temperature treatment, the most probable pore size of HDC is 40.3 nm.

The effect of different temperatures on harmless pores (<20 nm), less harmful pores (20 nm–50 nm), harmful pores (50 nm–200 nm), and more harmful pores (>200 nm) in HDC is analyzed [34]. As shown in Figure 18c, within the temperature range of −30 °C~60 °C, with the increase in temperature, the porosity of harmless pores and less harmful pores gradually increases, while the porosity of harmful pores and more harmful pores gradually decreases.

As the temperature increases, the hydration rate of the cementitious materials in HDC increases, and the hydration products become denser, causing harmful and more harmful pores in HDC to gradually transform into harmless and less harmful pores, leading to an increase in the porosity of harmless and less harmful pores in HDC, which, in turn, results in a decrease in critical pore size and the most probable pore size. As the temperature increases, the pore structure becomes denser [35], resulting in an increasing trend in the compressive strength, ultimate tensile strength, and flexural strength of HDC.

In this research, the effect of ambient temperature on the mechanical properties of HDC subjected to 8 h of exposure is analyzed. The sensitivity property index related to the ambient temperature is determined, such as the ultimate tensile strength, ultimate tensile strain, flexural strength, and flexural deflection. As HDC bridge deck link slabs are exposed to the natural environment for several years, a longer period reflecting the service life of link slabs will be considered in further research. The study in this paper can provide the basis for temperature effects on the tensile and flexural properties of HDC exposed for a longer period in future work.

## 4. Conclusions

This study was conducted to obtain the mechanical property parameters for the structural design of HDC bridge deck link slabs. The compressive, tensile, and flexural performances of HDC exposed to five temperature treatments (20, −30, 0, 40, and 60 °C) and the pore structure of HDC were analyzed.

1. Compared with HDC treated at room temperature (20 °C), the cubic compressive strength, axial compressive strength, and elastic modulus of HDC treated at low temperatures (−30 and 0 °C) decrease, while those treated at high temperatures (40 and 60 °C) increase. The Poisson’s ratio of HDC remained almost constant after treatment at different temperatures. In the design of HDC bridge deck link slabs, the compressive strength indicators of HDC under room temperature can be used without considering the temperature effect.
2. After treatment at different temperatures, multiple cracking is observed in the tensile specimens of HDC, and the stress–strain curves show strain-hardening stages. The constitutive relationship can be simplified as a bilinear model. Compared with HDC treated at room temperature (20 °C), the ultimate tensile strength of HDC decreases after treatment at low temperatures (−30 and 0 °C), while it increases after treatment at high temperatures (40 and 60 °C). The ultimate tensile strain and strain energy of HDC increase after treatment at low and high temperatures. In the design of HDC bridge deck link slabs, the effect of temperature on the tensile properties of HDC should be considered.
3. HDC exhibits deflection hardening in its flexural stress–deflection relationship, and the failure mode is multiple cracking. After subjecting the material to low temperatures (−30 and 0 °C), the flexural strength of HDC decreases, while the ultimate deflection and energy absorption increase, compared to its performance at room temperature (20 °C). However, after being exposed to high temperatures (40 and 60 °C), the flexural strength, ultimate deflection, and energy absorption of HDC increase. When analyzing the structural property of HDC bridge deck link slabs, it is important to consider the influence of temperature on the flexural property parameters of HDC.
4. The porosity of harmless and less harmful pores in HDC gradually increases as the temperature increases within the range of −30 to 60 °C, while the porosity of harmful and more harmful pores decreases. After being treated at a high temperature of 60 °C, both the critical pore size and most probable pore size of HDC decrease.

## Figures and Tables

**Table 1:** Mix proportion of HDC (kg/m^3^).

Specimen ID	C	FA	S	PS	W	PVA
HDC-1	680	600	384	1.01	384	26
HDC-2	768	512	384	1.54	397	26
HDC-3	768	512	384	1.31	323	26
HDC-4	768	512	384	1.92	253	26

**Table 2:** Dimensions and amounts of HDC specimens.

Test Program	Dimension/(mm × mm × mm)	Amount
Cubic compressive strength	100 × 100 × 100	3
Uniaxial tensile property	13 × 30 × 100 (Dimension of pure tension zone)	6

**Table 3:** Dimensions and amounts of HDC specimens.

Test Program	Dimension/(mm × mm × mm)	Amount
Cubic compressive strength	100 × 100 × 100	3
Axial compressive property	100 × 100 × 300	3
Uniaxial tensile property	13 × 30 × 100 (Dimension of pure tension zone)	6
Flexural property	15 × 75 × 300	3

**Table 4:** Mechanical properties of four different HDC mixtures.

Specimen ID	Cubic Compressive Strength/MPa	Ultimate Tensile Strength /MPa	Ultimate Tensile Strain /%
HDC-1	48.0 ± 0.99	4.23 ± 0.09	1.15 ± 0.05
HDC-2	47.8 ± 2.4	4.47 ± 0.15	0.64 ± 0.07
HDC-3	50.2 ± 3.2	4.22 ± 0.23	0.55 ± 0.04
HDC-4	59.0 ± 1.9	4.83 ± 0.17	0.42 ± 0.05

**Table 5:** Feature parameters of compressive property for HDC.

Temperature /°C	Cubic Compressive Strength $f_{cu}$/MPa	Axial Compressive Strength $f_{c}$/MPa	Elastic Modulus /GPa	Poisson’s Ratio	fcfcu
−30	46.7 ± 1.02 (−2.71%)	42.0 ± 1.11 (−2.55%)	23.2 ± 0.6 (−2.52%)	0.23	0.90
0	47.1 ± 1.13 (−1.88%)	42.5 ± 1.23 (−1.41%)	23.5 ± 0.7 (−1.26%)	0.23	0.90
20	48.0 ± 0.99 (~)	43.1 ± 0.98 (~)	23.8 ± 0.8 (~)	0.23	0.90
40	50.9 ± 1.15 (6.04%)	45.7 ± 1.18 (6.03%)	24.0 ± 0.98 (0.84%)	0.23	0.90
60	52.7 ± 1.19 (9.79%)	47.6 ± 1.32 (10.44%)	24.1 ± 0.88 (1.26%)	0.23	0.90

Notes: value in () means the increased amplitude of property indicator after low- or high-temperature treatment compared with that after room temperature treatment.

**Table 6:** Feature parameters of tensile property for HDC.

Temperature /°C	Ultimate Tensile Strength $\sigma_{tu}$/MPa	Ultimate Tensile Strain $\varepsilon_{tu}$/%	Strain Energy /(J/m^3^ × 10^6^)
−30	3.74 ± 0.08 (−11.58%)	1.34 ± 0.06 (16.52%)	4.42 (6.76%)
0	3.99 ± 0.10 (−5.67%)	1.46 ± 0.08 (26.96%)	4.76 (14.98%)
20	4.23 ± 0.09 (~)	1.15 ± 0.05 (~)	4.14 (~)
40	4.46 ± 0.08 (5.44%)	1.23 ± 0.07 (6.96%)	4.76 (14.98%)
60	4.80 ± 0.09 (13.48%)	1.31 ± 0.06 (13.91%)	5.14 (24.15%)

Notes: value in () means the increased amplitude of property indicator after low- or high-temperature treatments compared with that after room temperature treatment.

**Table 7:** Calculation parameters of simplified model of tensile stress–strain relationship for HDC.

Temperature /°C	Elastic Stage		Strain-Hardening Stage			
	$E_{tc}$/MPa	$R^{2}$	$E_{tu}$/MPa	$R^{2}$	$\sigma_{ntc}$/MPa	$\varepsilon_{ntc}$/%
−30	11.16 × 10^3^	0.94	79.91	0.94	2.79	0.025
0	16.76 × 10^3^	0.98	69.55	0.84	1.56	0.014
20	10.21 × 10^3^	0.99	60.70	0.81	2.86	0.028
40	15.62 × 10^3^	0.99	57.82	0.80	3.28	0.021
60	13.89 × 10^3^	0.99	95.43	0.88	2.92	0.021

**Table 8:** Feature parameters of flexural property for HDC.

Temperature /°C	Ultimate Load /kN	Flexural Strength /MPa	Ultimate Deflection /mm	Energy Absorption /J
−30	0.86 ± 0.32 (−13.13%)	12.23 ± 0.32 (−13.13%)	4.75 ± 0.15 (33.80%)	3.45 (25.45%)
0	0.92 ± 0.38 (−7.07%)	13.08 ± 0.38 (−7.07%)	5.01 ± 0.16 (41.13%)	3.95 (43.64%)
20	0.99 ± 0.34 (~)	14.08 ± 0.34 (~)	3.55 ± 0.13 (~)	2.75 (~)
40	1.03 ± 0.28 (4.04%)	14.65 ± 0.28 (4.04%)	3.82 ± 0.15 (7.61%)	3.25 (18.18%)
60	1.10 ± 0.38 (11.11%)	15.64 ± 0.38 (11.11%)	4.19 ± 0.17 (18.03%)	3.52 (28.00%)

Notes: value in () means the increased amplitude of property indicator after low- or high-temperature treatments compared with that after room temperature treatment.

## Data Availability

The data presented in this study are available on request from the corresponding authors.
